# An Account of the Efficacy of Arsenic in Intermittents

**Published:** 1788

**Authors:** J. C. Jenner

**Affiliations:** Surgeon at Painswick, in Gloucestershire


IV. *An Account of the Efficacy of Arfenic in Intermittents. Communicated in a Letter to Dr.* Simmons *by Mr.* J. C. Jenner, *Surgeon at Painfwick, in Gloucefterfhire.*

THIS town, which is fituated on the fide of a hill, and is remarkable for the purity of its air, is very populous. In the year 1784 the epidemic ague, that prevailed in many parts of the kingdom, made its appearance in this place, and has continued till the prefent time; although previoufly to that period the difeafe was hardly ever feen here, unlefs a ftranger came with it, for the recovery of his health, on account of the healthy fituation of the place.

It affected whole families, and appeared to be moft violent in fpring and autumn. In the fummer of 1786 it was followed by a fever of the kind kind called typhus, or low nervous, which not unfrequently degenerated into a putrid fever, and proved very fatal.

For the cure of the intermittents the bark was adminiftered in a variety of forms and dofes. Some were entirely cured by it; but in the greater number of patients the difeafe returned, and continued for many months. It was, indeed, feldom fufpended any longer than the ufe of the bark was continued; and as the difeafe was chiefly amongft the poor, many of whom could not afford to take fo expenfive a medicine long enough, the difeafe, in fuch cafes, continued till the conftitution either got the better of it, or till it brought on other complaints, and deftroyed the patient. Similar events alfo took place in patients who, from vulgar prejudice, refufed to take the bark; and in children who could not be prevailed with to take it in a proper quantity.

About this time Dr. Fowler having publifhed his Medical Reports of the Effects of Arfenic in Agues, &c. I began to make trial of its effects, and I am happy in being able to fay that it has never failed me of fuccefs where it has been taken properly, and no collateral circumftances have forbid the ufe of it. I have now given it to upwards of two hundred patients, and have never feen any bad effects from its adminiftration; and I think it is not improbable that in the cafes where it is faid to have produced violent effects, (and which have deterred many perfons from having recourfe to it) fome particle of arfenic has got into the ftomach in an undiffolved ftate, for want of the medicine being carefully filtered after it is cold, and the whole quantity of water added; a precaution Dr. Fowler has omitted, but which I think abfolutely neceffary to the fafe adminiftration of the folution. I have given it in very large dofes, and have never feen any alarming fymptom from a cautious adminiftration of it. In one patient, however, who, by miftake, took twenty-five inftead of fifteen drops * of the folution at a dofe, for the cure of an intermittent, it brought on a fwelling of the face; but this went off on her leaving off the medicine, and the patient continues free from ague and every other complaint. With another patient, a poor man who had had a quotidian fifteen months, and had taken large quantities of bark, as well as

* Eighty drops of the folution contain about half a grain of arfenic. — See Vol. VII. page 197.

other other medicines, for the cure of it, but without ſuſpending it longer than a few days, I began with fifteen drops of the mineral ſolution three times a day. At the end of five days, as it produced no good effect, I increaſed the doſe to eighteen drops, which he continued three days, but ſtill without effect. The medicine was then omitted for three or four days, and then given again in doſes firſt of twenty, and afterwards of twenty-five drops three times a day. In this laſt doſe it produced griping and pain of the bowels, with diarrhœa, but put a ſtop to the diſeaſe. I now omitted the ſolution, gave him an ounce of tincture of rhubarb in the morning, and an opiate at night, put him on a milk diet, and, in a few days, the complaint of his bowels ceaſed, and he has remained well ever ſince.

As a proof of the efficacy of the mineral ſolution in a violent periodical head-ach, I ſhall add the following caſe:

In the beginning of June, 1787, a gentleman, aged twenty-one years, and of a delicate conſtitution, was attacked with a pain of his head, attended with nauſea. An emetic afforded him ſome relief; but as the head-ach continued to return every night, he had recourſe to the bark, and the complaint ceafed. In a few days, however, it returned again with great violence, affecting principally the forehead and one fide of the face and teeth. A carious tooth was now extracted at the earneft defire of the patient; but the pain ftill continued, accompanied with fpafmodic affections of various parts. After this, by the advice of different phyficians of eminence, who were confulted at different times, he tried a variety of remedies both topical and internal. Among the former were leeches, blifters, cupping, fomentations, and æther. Æther was alfo adminiftered internally. Opium, with camphor and antimonial wine, were given during the paroxyfms, and in the intermiffions the bark was exhibited freely. The opiate alleviated, in fome meafure, the violence of the pain, and the bark repeatedly fufpended the complaint for feveral days; but it returned again even during the ufe of the remedy, and was at times fo violent that the patient cried out; and, in order to leffen it, was fometimes obliged to have his head preffed the whole night by an attendant.

At the beginning of September, the pain ftill continuing to return, and the bark (of which he had tried both the red and the pale fpecies, and was ftill taking to the amount of an ounce a day in fubftance) being now become offenfive to his ftomach, and procuring no relief, I determined to try the mineral folution. Fifteen drops of it were accordingly given thrice a day for five days, and during this period the difeafe gradually difappeared. The medicine produced no griping or pain of the bowels, nor any other fenfible effect, excepting a flight naufea after the morning dofe, which was probably owing to the emptinefs of the ftomach. It was obferved, however, that his appetite was rather impaired during the ufe of the folution, a circumftance that had never happened before during the whole courfe of the difeafe. It was, therefore, omitted, and in its ftead two teafpoonfuls of a mixture of equal parts of Huxham's tincture of bark and the fpirituous tincture of rhubarb were directed to be taken. At the end of a week the folution was again repeated as before, for three days, by way of prevention; but was then wholly laid afide, as he continued to be free from complaint, and has remained well ever fince.

I could communicate to you a variety of other inftances of the falutary effects of this medicine; but thofe I have mentioned, in addition to the cafes publifhed by Dr. Fowler in the work already referred to, and by Dr. Willan in the London Medical Journal *, are fufficient to fhow that it is capable of curing the moft obftinate intermittents after the bark has had a fair trial and has failed of fuccefs.

*Painfwick,*

Dec. 14th, 1787.

